# Essential Roles of Heparan Sulfate Endosulfatase Sulf1 in Reward and Aversion Learning Through Distinct Dopamine D1 and D2 Receptor Pathways in Male Mice

**DOI:** 10.1111/jnc.70338

**Published:** 2026-01-04

**Authors:** Ken Miya, Kent Ohta, Kazuko Keino‐Masu, Takuya Okada, Seiya Mizuno, Satoru Takahashi, Tom Macpherson, Takatoshi Hikida, Masayuki Masu

**Affiliations:** ^1^ Graduate School of Comprehensive Human Sciences University of Tsukuba Tsukuba Ibaraki Japan; ^2^ Department of Molecular Neurobiology, Institute of Medicine University of Tsukuba Tsukuba Ibaraki Japan; ^3^ Laboratory for Advanced Brain Functions, Institute for Protein Research The University of Osaka Suita Osaka Japan; ^4^ Graduate School of Medicine The University of Osaka Suita Osaka Japan; ^5^ Laboratory Animal Resource Center, Transborder Medical Research Center, Institute of Medicine University of Tsukuba Tsukuba Ibaraki Japan; ^6^ Graduate School and Faculty of Pharmaceutical Sciences Kyoto University Kyoto Kyoto Japan

**Keywords:** conditioned place preference test, heparan sulfate, inhibitory avoidance test, knockout mouse, medium spiny neuron, nucleus accumbens, Sulf1

## Abstract

Sulf1 and Sulf2 are extracellular sulfatases that remove 6‐*O*‐sulfate from heparan sulfate and thereby regulate cell signaling. Previous studies have revealed that *Sulf1/Sulf2* double knockout (KO) mice had defects in differentiation and axon guidance during development, but their functional roles in the adult brain remain largely unknown. We recently found that *Sulf1* mRNA is highly expressed in the nucleus accumbens (NAc) shell and that *Sulf1* expression is detected in both types of medium spiny neurons expressing dopamine D1 or D2 receptors. Moreover, we found that *Sulf1* KO led to changes in membrane excitability and excitatory synaptic transmission in medium spiny neurons of the NAc in adult mice. These findings suggest possible roles of *Sulf1* in the functions of NAc circuitry. To address this question, we performed behavioral tests using *Sulf1* KO mice. We found that constitutive *Sulf1* KO mice showed impairment in both the cocaine‐induced conditioned place preference (CPP) test and inhibitory avoidance (IA) test. Next, to examine which cell types the *Sulf1* gene is required for, we generated *Sulf1* floxed mice by means of CRISPR‐Cas9‐mediated genome editing and mated them with mice expressing Cre recombinase under a promoter for either the dopamine D1 or D2 receptor‐encoding genes. *Sulf1* conditional knockout (cKO) in cells expressing dopamine D1 receptors led to impairment only in the CPP test, whereas *Sulf1* cKO in D2 receptor‐expressing cells resulted in impairment only in the IA test. These results demonstrate that *Sulf1* is required for both reward and aversion learning, and that the D1‐ and D2‐pathways distinctly regulate these functions. The present study suggests that *Sulf1* is essential for neuronal functions and behavioral control in the adult brain.

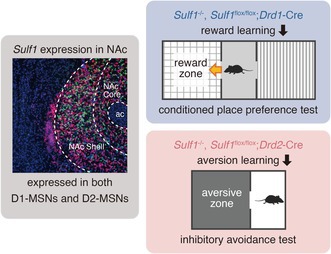

## Graphical abstract

In the nucleus accumbens (NAc), a heparan sulfate endosulfatase *Sulf1* is expressed in both dopamine D1 receptor (*Drd1*) expressing and *Drd2*‐expressing medium spiny neurons (D1‐MSNs and D2‐MSNs, respectively). *Sulf1* knockout (KO) mice showed impairment in both the cocaine‐induced conditioned place preference (CPP) test and inhibitory avoidance (IA) test. Conditional *Sulf1* disruption in *Drd1*‐ and *Drd2*‐expressing cells led to impairment in the CPP test and IA test, respectively. These results demonstrated that *Sulf1* is required for both reward and aversion learning, and that the D1‐ and D2‐pathways distinctly regulate these functions.

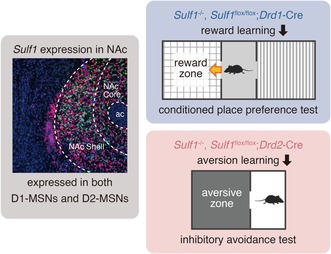

**Abbreviations**
ANOVA: analysis of variance
cKO: conditional knockout
CPP: conditioned place preference
D1R: dopamine D1 receptor
D2R: dopamine D2 receptor
Drd1: dopamine D1 receptor (gene)
Drd2: dopamine D2 receptor (gene)
ECM: extracellular matrix
HS: heparan sulfate
HSPG: heparan sulfate proteoglycan
IA: inhibitory avoidance
KO: knockout
MSN: medium spiny neuron
NAc: nucleus accumbens
PBS: phosphate buffered saline
PFC: prefrontal cortex
PVT: paraventricular nucleus of the thalamus
ROI: region of interest
RRID: Research Resource Identifier
RT‐PCR: reverse transcription polymerase chain reaction
Sulf1: sulfatase 1
TS: tail of the striatum
VP: ventral pallidum
VTA: ventral tegmental area
WT: wild‐type

## 1 Introduction

Heparan sulfate proteoglycans (HSPGs) are glycoproteins present ubiquitously on the cell surface and in the extracellular matrix (ECM). They regulate cell differentiation, proliferation, migration, axon guidance, synaptogenesis, and synaptic plasticity in the nervous system (Holt and Dickson 2005; Masu 2016; Condomitti and de Wit 2018; Kamimura and Maeda 2021). They consist of core proteins and covalently attached heparan sulfate (HS) chains. HS is a linear polysaccharide that consists of repeating disaccharides composed of glucuronic or iduronic acid and *N*‐acetylglucosamine. During biosynthesis, HS undergoes extensive sulfation at the *N*‐, 6‐*O*, and 3‐*O* positions of glucosamines and the 2‐*O* position of glucuronic/iduronic acids. HSPGs exert a wide variety of biological functions through interaction with various signaling molecules, including growth factors, cytokines, receptors, enzymes, and ECM molecules via HS chains. The degree and specific positions of sulfate groups in the HS are crucial determinants for specificity and affinity for the interaction with binding proteins (Perrimon and Bernfield 2000; Bishop et al. 2007; Masu 2016).

Sulfatase 1 (Sulf1) and Sulfatase 2 (Sulf2) are HS endosulfatases that remove 6‐*O*‐sulfate from the HS extracellularly and thereby regulate cellular signaling positively or negatively (Dhoot et al. 2001; Morimoto‐Tomita et al. 2002; Lamanna et al. 2007; El Masri et al. 2017). Previous studies have revealed that *Sulf1/2* play important roles in the regulation of neural differentiation, axon guidance, and skeletal and renal abnormalities during development (Ai et al. 2007; Holst et al. 2007; Okada et al. 2017), but their roles in the adult brain remain largely unknown. We thus examined the expression patterns of *Sulf1* and *Sulf2* mRNAs in the adult mouse brain systematically and found that *Sulf1* is abundantly expressed in the nucleus accumbens (NAc), prefrontal cortex (PFC), tail of the striatum (TS), and paraventricular nucleus of the thalamus (PVT) (Miya et al. 2021). *Sulf1* expression is colocalized with dopamine D1 receptor (*Drd1*) and dopamine D2 receptor (*Drd2*) expression in these regions (Miya et al. 2021). In contrast, *Sulf2* mRNA is expressed in astrocytes, but not in neurons, in the NAc (Miya et al. 2021). More recently, by performing electrophysiological analysis of *Sulf1* knockout (KO) mice, we found that *Sulf1* regulates membrane excitability and glutamatergic synaptic transmission in NAc neurons (Miya et al. 2025). These data strongly suggest the possibility that *Sulf1* is involved in brain functions mediated by the NAc in the adult mouse.

The NAc is a part of the ventral striatum and functions as a neural network hub in the corticolimbic circuitry. It plays a key role in motivation, reward processing, cognition, and associative learning (Kreitzer 2009; Macpherson et al. 2014; Floresco 2015; Castro and Bruchas 2019; Marinescu and Labouesse 2024; Vieitas‐Gaspar et al. 2025). It receives excitatory inputs from the PFC, ventral hippocampus, basolateral amygdala, and PVT, and dopaminergic inputs from the ventral tegmental area (VTA) that modulate the NAc's activity. Most neurons in the NAc (approximately 95%) are GABAergic medium spiny neurons (MSNs), which can be subdivided into two groups: D1‐MSNs expressing dopamine D1 receptors (D1R) and D2‐MSNs expressing the dopamine D2 receptors (D2R). A subpopulation of the MSNs expresses both D1R and D2R, with a reported frequency of approximately 2%–17% (Bertran‐Gonzalez et al. 2008; Kupchik et al. 2015; Gagnon et al. 2017; Miyasaka et al. 2025). D1‐MSNs project directly to the ventral mesencephalon (VM; the VTA and substantia nigra) and indirectly to the mediodorsal thalamus and VM via the ventral pallidum (VP), whereas D2‐MSNs project directly to the VP (Kupchik et al. 2015). Although many previous studies have dissected the roles of D1‐ and D2‐MSNs in the NAc by means of genetic approaches, it still remains unclear how NAc D1‐ and D2‐pathways are involved in reward‐ and aversion‐related behaviors. Moreover, the NAc is anatomically subdivided into two subregions, the core and the shell, and a large body of work has demonstrated that the NAc core and shell play distinct yet complementary roles in motivated behavior and reward processing (Zahm and Brog 1992; Di Chiara 2002; Kelley 2004; Castro and Bruchas 2019; Marinescu and Labouesse 2024; Vieitas‐Gapar et al., 2025).

In this study, to explore the in vivo roles of *Sulf1* in the adult brain, we performed behavioral tests using *Sulf1* KO mice. We show that constitutive *Sulf1* disruption leads to impairment in both the cocaine‐induced conditioned place preference (CPP) test and inhibitory avoidance (IA) test. Furthermore, by using *Sulf1* conditional knockout (cKO) mice, we demonstrate that disruption of the *Sulf1* gene in D1R‐ and D2R‐expressing cells results in selective impairment of the CPP and IA tests, respectively. Our data suggest that *Sulf1* is required for reward and aversion learning in distinct neuronal pathways.

## 2 Materials and Methods

### 2.1 Animals

Three‐ to 5‐month‐old male mice were used in this study. The total number of the mice used was 124. The number of animals used for each experiment is described in the corresponding figure legends or text. All animal experiments were approved by and performed according to the guidelines of the Animal Care and Use Committees of University of Tsukuba and the University of Osaka (approval numbers #25–252, #25–256, and R04‐01‐2). Mice were maintained under standard conditions: 12 h light/dark cycle (light on from 8 a.m. to 8 p.m.); room temperature 23.5°C ± 2.5°C (University of Tsukuba) and 24°C ± 2°C (the University of Osaka); and humidity at 52.5% ± 12.5% (University of Tsukuba) and 50% ± 5% (the University of Osaka). They were housed in groups of 2–5 individuals in standard plastic cages with *ad libitum* access to standard laboratory chow and water. Body weights of mice used were 25–30 g. The mouse strains used were *Sulf1* KO mice (MGI 5318489; Nagamine et al. 2012), *Drd1*‐*Cre* mice (MGI 4358480), *Drd2*‐*Cre* mice (MGI 5003554), and *Nestin*‐*Cre* mice (MGI 2176222). *Sulf1* KO mice were generated in our laboratory; *Drd1*‐*Cre* and *Drd2*‐*Cre* mice were purchased from Mutant Mouse Resource and Research Center (MMRC); and *Nestin*‐*Cre* mice were provided by Dr. Kageyama (Kyoto University). All mice were backcrossed to C57BL/6N for 10 successive generations. Genotypes were determined by genomic PCR with tail biopsy by use of KOD FX Neo or KOD ONE (TOYOBO, Osaka, Japan). The experimenters were not blinded to the genotypes of mice except as otherwise mentioned.

### 2.2 Generation of a *Sulf1* Floxed Mouse

*Sulf1* floxed mice were generated by inserting two loxP sites flanking exons 6 and 7 of the *Sulf1* gene by means of a CRISPR‐Cas9‐mediated genome editing strategy (Hsu et al. 2014; see Figure 3). Briefly, a donor vector pflox‐*Sulf1* was constructed by inserting three genomic sequences of the *Sulf1* gene: the 5′ region (0.6–2.4 kb upstream of exon 6; 1761 bp), the center region (0.6 kb upstream of exon 6 to 1.0 kb downstream of exon 7; 4435 bp), and the 3′ region (1.0–2.9 kb downstream of exon 7; 1900 bp) into the PmeI, AscI, and NotI sites of the pflox vector (Nakagawa et al. 2016), respectively, by use of the In‐fusion HD cloning kit (Takara Bio, Kusatsu, Japan). The donor vector pflox‐*Sulf1*, single‐guided RNAs that correspond to two target sequences (5′‐GAAGGCGCATTAGAGCTACA‐3′ and 5′‐CAGGTATTGTGCATCCCGCG‐3′), and the pX330‐mG plasmid carrying Cas9 expression sequence (Nguyen et al. 2025) were co‐injected into male pronuclei of fertilized eggs of C57BL/6N mice. Among 35 mice produced by in vitro fertilization, three individuals contained two loxP sites on the same allele. Genomic PCR confirmed that none of them contained the sequences of the ampicillin resistance gene from the donor vector and pX330 plasmid nor the Cas9 gene from the pX330‐mG plasmid. When the *Sulf1* floxed mice were mated with *Nestin*‐*Cre* mice, we found that Cre‐mediated recombination occurred in the germline rather than specifically in the brain at a low frequency as reported previously (Luo et al. 2020; McLeod et al. 2020). Thus, mice with germline Cre‐mediated excision of *Sulf1* exons 6–7 were used as carriers of the *Sulf1*‐excised allele.

### 2.3 Reverse Transcription Polymerase Chain Reaction (RT‐PCR)

Male mice (18–19 weeks old) were decapitated immediately after cervical dislocation and their brains were removed. Total RNAs were extracted from whole brains using RNAiso Plus (Takara Bio) and purified using an RNeasy kit (Qiagen, Hilden, Germany). The cDNAs were generated by use of Superscript III and oligo(dT)_12–18_ primers (Thermo Fisher Scientific, Waltham, MA, USA). The cDNAs were amplified by AmpliTaq Gold DNA polymerase (Thermo Fisher Scientific) and specific primers listed in Table 1.

**TABLE 1:** Primers for PCR.

Primer name	Sequence (5′—3′)	Purpose	Amplified length (primer combination)	Figures
Sulf1 cKO step1seq4F (#1)	ACAGAGGTTCATTGCTCTAGTC	Genotyping (Sulf1‐flox)	WT, ~5.1 kb; Excised, ~0.7 kb (#1 & #2)	Figure 3b,c, designated as F1
Sulf1 cKO step3seq8R (#2)	GAGCCACCTGCTACTAGGATTC	Genotyping (Sulf1‐flox)		Figure 3b,c, designated as R1
Sulf1 exon4F (#3)	AAGTATTCCCTCTGGGCTCTG	RT‐PCR	WT, 456 bp (#3 & #4)	Figure 3e
Sulf1 exon6R (#4)	AGGGATGTAGCTGCCATTGTAT	RT‐PCR		Figure 3e
Sulf1 exon5 Left (#5)	CACCAATGCCTTTGTGACC	RT‐PCR	WT, 959 bp; Excised, 637 bp (#5 & #6)	Figure 3d
Sulf1exon10R (#6)	TGTTACCCGGCTTTTCCAGGTC	RT‐PCR		Figure 3d
Drd1a F1 (#)	GCTATGGAGATGCTCCTGATGGAA	Genotyping (Drd1‐Cre)	Tg, 340 bp (#7 & #9)	
Drd2 F1 (#8)	GTGCGTCAGCATTTGGAGCAA	Genotyping (Drd2‐Cre)	Tg, 700 bp (#8 & #9)	
CreGS R1 (#9)	CGGCAAACGGACAGAAGCATT	Genotyping (Drd1‐Cre, Drd2‐Cre)		

### 2.4 Multiplex Fluorescence In Situ Hybridization

Male mice (13–14 weeks old) were deeply anesthetized by an open‐drop method of isoflurane exposure. After confirming the loss of the righting or tail‐pinch reflex, they were transcardially perfused with 4% paraformaldehyde in phosphate buffered saline (PBS). Brains were dissected, postfixed with the same solution at 4°C overnight, cryoprotected in 30% sucrose/PBS, embedded in Tissue‐Tek OCT compound (Sakura Finetek Japan, Tokyo, Japan), and stored at −80°C. Coronal brain sections (14‐μm thick) were cut by means of a cryostat (CM 1850; Leica Biosystems, Wetzlar, Germany) and collected onto MAS‐coated slide glasses (Matsunami Glass Industry, Osaka, Japan).

Multiplex fluorescence in situ hybridization was performed by use of an RNAscope Multiplex Fluorescent v2 Assay kit (Advanced Cell Diagnostics, Hayward, CA, USA) following the manufacturer's instructions. For detection of *Drd1* and *Drd2* mRNA, the specific target probes for *Drd1* (Mm‐Drd1a‐C2, #406491‐C2) and *Drd2* (Mm‐Drd2‐C3, #406501‐C3) were used. For detection of *Sulf1* mRNA, a *Sulf1*‐specific probe set (Mm‐Sulf1‐O1‐C1, #1807841‐C1) was custom‐designed: it includes the 322‐bp sequence corresponding to exons 6–7 and approximately 50‐bp overhang extension each in exons 5 and 8 to meet the length required for obtaining sufficient signals according to the manufacturer's recommendation. Signals for *Sulf1*, *Drd1*, and *Drd2* were developed with TSA Vivid Fluorophore kits 650, 570, and 520 (Tocris Biosciences, Bristol, UK), respectively. The sections were mounted with CC/Mount (Diagnostic BioSystems, Pleasanton, CA, United States).

### 2.5 Image Analysis

Microscopic images were obtained and processed under the same conditions for all samples using the method described below. Z‐stacking (1‐μm interval) images were acquired by use of a laser scanning confocal microscopy (LSM 700; Carl Zeiss, Jena, Germany) with a 20× objective lens and 2× digital zoom. Two regions of interest (ROIs, 160‐μm square) were selected in the NAc shell regions from each of the 3 mice. To analyze colocalization of *Sulf1* and *Drd1/Drd2*, cells positive for *Sulf1*, *Drd1*, and *Drd2* were marked manually and overlapping was analyzed as follows: by an expert who was blinded to the genotypes of the specimens. Because strong signals were obtained for *Drd1* and *Drd2*, it was easy to determine the positive cells in the original images, but in the case of *Sulf1*, it was difficult to distinguish weak positive signals from the background signals. Therefore, the *Sulf1*‐positive cells were determined by using the following procedure. First, the image files were opened in the 3D view of Imaris software (RRID:SCR_007370; Bitplane, Zürich, Switzerland) and the magenta channel images for *Sulf1* were acquired using the Snapshot function. Next, the files were opened in ImageJ Fiji (RRID:SCR_002285; https://imagej.net/ij/), and all the particles in the images were identified by setting an appropriate threshold, and the mean brightness values for each particle were obtained by means of the Analyze Particle function. When the brightness values in all specimens were examined, there were many particles with brightness values of 45 or less, corresponding to approximately 74% of all particles (Figure S1). As these weak signals appeared to be background signals, we decided to remove them from the *Sulf1* images to clearly display only the positive signals. For this purpose, the original files were opened by setting the threshold of the magenta channel (for *Sulf1*) to 45, and the cells positive for *Sulf1*, *Drd1*, or *Drd2* were marked independently in each of the three channels: for *Sulf1*, cells in which at least one dot was detected in the cell body (on or immediately adjacent to the nuclear DAPI signals) were counted as positive (Figure S1), whereas for *Drd1* and *Drd2*, cells that had sufficiently strong signals in the cell body were counted as positive. Finally, by integrating the three channels, we investigated whether each cell was positive for *Sulf1*, *Drd1*, and *Drd2* expression and analyzed their colocalization.

### 2.6 Conditioned Place Preference (CPP) Test

Cocaine‐induced CPP test was performed using 12‐ to 16‐week‐old mice as described previously (Hikida et al. 2010). A three‐compartment apparatus (ENV‐3013; MED Associates, Fairfax, VT, USA), composed of a white chamber with a white mesh floor, a black chamber with a black grid rod floor, and a gray center compartment connecting the two chambers, was used. Animal position is tracked by means of infrared photobeam detectors. On Day 1, mice were allowed to move freely and access all three compartments for 30 min. On Days 2–4, mice were injected intraperitoneally with cocaine (5 mg/kg) or saline and confined to one of the large chambers for 20 min. Four hours later, they were given the alternate treatment and confined to the opposite large chamber for 20 min. On Day 5, the mice were again allowed to move freely and access all three compartments for 30 min. A 5 mg/kg dose of cocaine was selected based on previous reports (Tilley et al. 2009; Rogge et al. 2013; Chesworth et al. 2023). We used an unbiased design, in which the assignment of the chamber paired with cocaine is determined regardless of the preference of a mouse prior to the conditioning. The order of cocaine/saline injections and the chamber in which mice were placed after cocaine/saline administration were counterbalanced. No randomization was performed to allocate mice. Time (in s) spent in the cocaine‐paired chamber (Time C) and that in the saline‐paired chamber (Time S) was measured on Day 1 and Day 5, and place preference (Time C minus Time S, designated as “Time C − S”) was calculated. Mice displaying unconditioned preference (the absolute value of Time C − S on Day 1 was greater than 360 s) were excluded from subsequent experiments. The numbers of mice excluded were 3 in both the control and KO groups in the constitutive *Sulf1* KO mice, 3 in both the control and KO mice in D1 cKO mice, and 1 in KO mice in D2 cKO mice.

### 2.7 Inhibitory Avoidance (IA) Test

The IA test was performed using 12‐ to 16‐week‐old mice as previously described (Macpherson et al. 2019). A shuttle box (ENV‐3013, ENV‐414S; MED Associates) composed of a brightly lit chamber (180 lx) and a dark chamber that are linked through a sliding door was used. On the training day, mice were placed in the bright chamber. When the mice stepped into the dark chamber (when all four paws entered), the sliding door was closed and an electrical stimulation (0.3 mA, 60 Hz, 1 s) was provided from the metal grid floor. After the electrical stimulation, the mice were kept in the dark chamber for 1 min. Twenty‐four hours later, mice were again placed in the bright chamber and the step‐through latency from the lit chamber to the dark chamber (in s) was measured. When the step‐through latency was larger than 300 s on the test day, it was counted as 300 s. No mice were excluded from the analysis.

### 2.8 Statistical Analysis

No statistical methods were used to predict the sample size before the study. We determined the sample size based on our previous study (Hikida et al. 2010). The normality of data was verified using Quantile‐Quantile (Q‐Q) plots. No tests for outliers were performed. We calculated effect sizes and included them in Table 2. The observed effect sizes were large (*η*_p_^2^ = 0.24–0.70), indicating that the sample size was sufficient to detect significant differences in this experimental setting. Statistical analyses were performed by two‐way mixed model ANOVA with Bonferroni post hoc tests using Prism 8.0 (RRID:SCR_002798; GraphPad Software, Boston, MA, USA). Statistical differences were considered significant at *p* < 0.05. All statistical values are described in the figure legends and listed in Table 2.

**TABLE 2:** Results of the statistical analysis in behavioral experiments.

Figures	Data structure	Type of test	Sample size	Statistical data
Figure 1b	Normally distributed	Two‐way mixed model ANOVA followed by Bonferroni post hoc test	WT, *n* = 10 KO, *n* = 10	Genotype, *F*(1,18) = 5.83, *p* = 0.027, *η*_p_^2^ = 0.24
CPP test WT vs. Sulf1KO				Time, *F*(1,18) = 12.8, *p* = 0.0022, *η*_p_^2^ = 0.42
				Interaction, *F*(1,18) = 14.4, *p* = 0.0013, *η*_p_^2^ = 0.44
				Bonferroni post hoc test
				WT vs. KO
				Post‐test, *p* = 0.0002
Figure 2b	Normally distributed	Two‐way mixed model ANOVA followed by Bonferroni post hoc test	WT, *n* = 8 KO, *n* = 8	Genotype, *F*(1,14) = 8.63, *p* = 0.011, *η*_p_^2^ = 0.38
IA test WT vs. Sulf1KO				Time, *F*(1,14) = 7.87, *p* = 0.014, *η*_p_^2^ = 0.36
				Interaction, *F*(1,14) = 7.28, *p* = 0.017, *η*_p_^2^ = 0.34
				Bonferroni post hoc test
				WT vs. KO
				Retrieval, *p* = 0.0009
Figure 5a	Normally distributed	Two‐way mixed model ANOVA followed by Bonferroni post hoc test	Control, *n* = 8 D1cKO, *n* = 8	Genotype, *F*(1,14) = 6.22, *p* = 0.026, *η*_p_^2^ = 0.31
CPP test Control vs. D1cKO				Time, *F*(1,14) = 11.9, *p* = 0.0039, *η*_p_^2^ = 0.46
				Interaction, *F*(1,14) = 9.30, *p* = 0.0087, *η*_p_^2^ = 0.40
				Bonferroni post hoc test
				Control vs. D1cKO
				Post‐test, *p* = 0.0013
Figure 5b	Normally distributed	Two‐way mixed model ANOVA	Control, *n* = 6 D2cKO, *n* = 9	Genotype, *F*(1,13) = 0.699, *p* = 0.42
CPP test Control vs. D2cKO				Time, *F*(1,13) = 9.27, *p* = 0.0094, *η*_p_^2^ = 0.42
				Interaction, *F*(1,13) = 2.87, *p* = 0.11
Figure 6a	Normally distributed	Two‐way mixed model ANOVA	Control, *n* = 6 D2cKO, *n* = 6	Genotype, *F*(1,10) = 0.057, *p* = 0.82
IA test Control vs. D1cKO				Time, *F*(1,10) = 23.7, *p* = 0.0007, *η*_p_^2^ = 0.70
				Interaction, *F*(1,10) = 0.086, *p* = 0.78
Figure 6b	Normally distributed	Two‐way mixed model ANOVA followed by Bonferroni post hoc test	Control, *n* = 7 D2cKO, *n* = 7	Genotype, *F*(1,12) = 17.5, *p* = 0.0013, *η*_p_^2^ = 0.59
IA test Control vs. D2cKO				Time, *F*(1,12) = 17.9, *p* = 0.0012, *η*_p_^2^ = 0.60
				Interaction, *F*(1,12) = 13.5, *p* = 0.0032, *η*_p_^2^ = 0.53
				Bonferroni post hoc test
				Control vs. D2cKO
				Retrieval, *p* < 0.0001

## 3 Results

### 3.1 *Sufl1* Is Required for Both Reward and Aversion Learning

**FIGURE 1:**
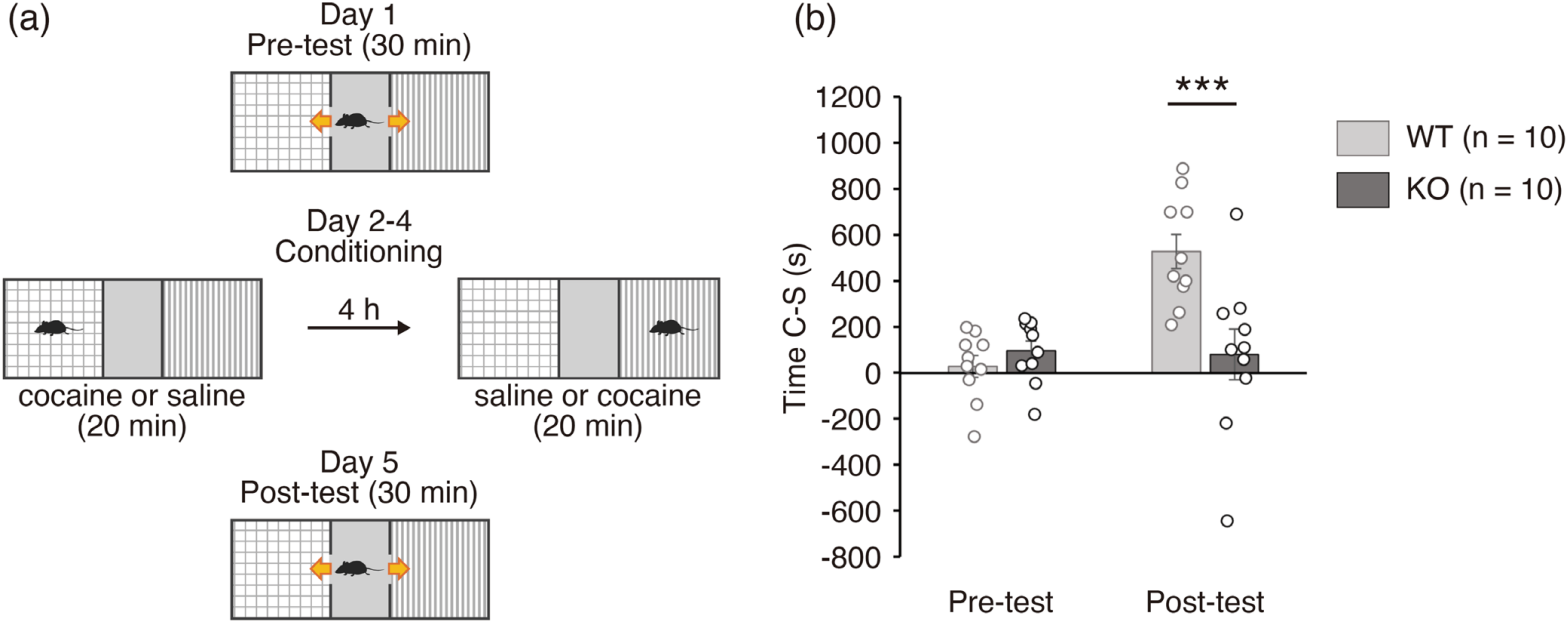
Learning in the conditioned place preference (CPP) test is impaired in *Sulfatase 1* (*Sulf1*) knockout (KO) mice. (a) Scheme of the CPP test. Time (s) spent in the cocaine‐paired chamber (Time C) and in the saline‐paired chamber (Time S) was measured before (Pre‐test) and after (Post‐test) cocaine conditioning (5 mg/kg). Time C minus Time S (Time C − S) is used as an indicator of place preference. See Materials and Methods for details. (b) The results of the CPP test. Time C − S was significantly lower in *Sulf1* KO mice (*n* = 10) than in wild‐type (WT) mice (*n* = 10) in the post‐test (main effect of genotype, *F*(1,18) = 5.83, *p* = 0.027; main effect of time, *F*(1,18) = 12.8, *p* = 0.0022; interaction, *F*(1,18) = 14.4, *p* = 0.0013; Bonferroni post hoc test, WT vs. KO in post‐test, *p* = 0.0002; two‐way mixed model analysis of variance [ANOVA]). Individual data points and means ± SEMs are shown. ***p < 0.001.

Given that the NAc plays important roles in reward and aversion learning, we examined whether the *Sulf1* gene is required for these associative learning by use of two behavioral tests. First, we performed a cocaine‐induced CPP test, which is often used as a measure of reward learning. On the first day, mice were allowed to move freely for 30 min in an apparatus composed of two chambers, which have different colored walls and different textured floors, that are connected with each other by a central compartment (Figure 1a). For the following 3 days, mice were confined to one of the two chambers for 20 min after intraperitoneal administration of cocaine (5 mg/kg) or saline, and 4 h later the mouse was confined to the opposite chamber after intraperitoneal administration of the alternate treatment. A 5 mg/kg dose of cocaine was selected based on previous reports showing robust CPP induction in mice without stereotypy (Tilley et al. 2009; Rogge et al. 2013; Chesworth et al. 2023). On the last day, mice were again allowed to move freely in the apparatus. Time spent in a cocaine‐paired chamber (Time C) and that in a saline‐paired chamber (Time S) were measured before and after cocaine conditioning, and the difference between Time C and Time S (Time C − S) was calculated as an indicator of place preference. As shown in Figure 1b, control wild‐type (WT) mice spent more time in the cocaine‐paired chamber in the post‐test than in the pre‐test, indicating that they showed a learned preference. In contrast, *Sulf1* KO mice did not show a clear preference after cocaine conditioning, and Time C − S was significantly lower in KO mice than in WT mice (Figure 1b). These data indicate that *Sulf1* is required for Pavlovian reward learning.

**FIGURE 2:**
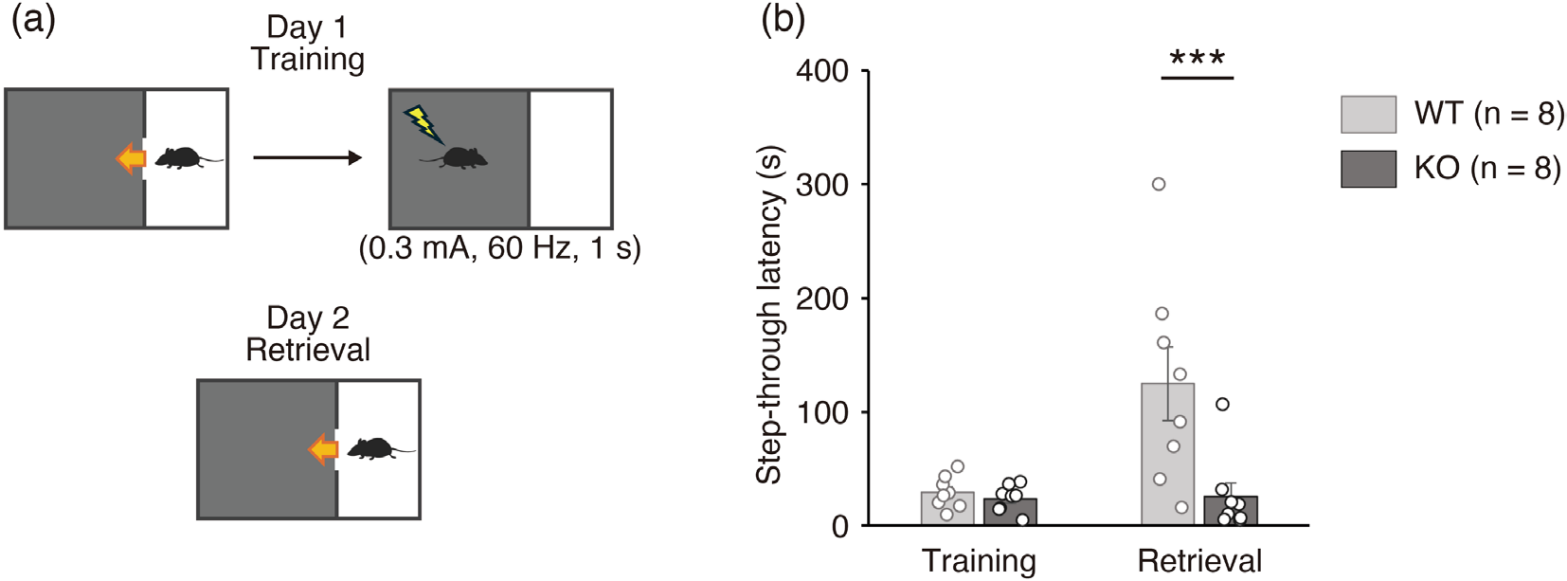
Learning in the inhibitory avoidance (IA) test is impaired in *Sulfatase 1* (*Sulf1*) knockout (KO) mice. (a) Scheme of the IA test. Step‐through latency (s) from the light chamber to the dark chamber was measured before (Training) and after (Retrieval) electrical shock (0.3 mA, 60 Hz, 1 s) in the dark chamber. See Materials and Methods for details. (b) The results of the IA test. Step‐through latency was significantly lower in *Sulf1* KO mice (*n* = 8) than in wild‐type (WT) mice (*n* = 8) after training (main effect of genotype, *F*(1,14) = 8.63, *p* = 0.011; main effect of time, *F*(1,14) = 7.87, *p* = 0.014; interaction, *F*(1,14) = 7.28, *p* = 0.017; Bonferroni post hoc test, WT vs. KO, Retrieval, *p* = 0.0009; two‐way mixed model analysis of variance [ANOVA]). Individual data points and means ± SEMs are shown. ***p < 0.001.

Next, we performed an IA test, also known as a passive avoidance test, to examine the role of *Sulf1* in aversion learning. In this test, mice are placed in a bright chamber of a device composed of a brightly lit chamber and a dark chamber (Figure 2a). When mice enter the dark chamber, the door connecting the two chambers is closed, an electrical shock is delivered, and the mice are kept in the dark room for 1 min. On the next day, mice are again placed in the bright chamber, and the step‐through latency from the bright chamber to the dark chamber is measured. As a preference for dark environments is an innate feature of mice, WT mice entered the dark room with a short latency before the training (Figure 2b). However, after having experienced an electrical shock in the dark chamber, control WT mice showed an increased latency to enter the dark chamber, suggesting that they learned to avoid the aversive stimuli (Figure 2b). In contrast, *Sulf1* KO mice did not show an extended latency after training, and the latency was significantly shorter in KO mice than in WT mice (Figure 2b). These data indicate that *Sulf1* is also required for Pavlovian aversive learning.

### 3.2 Generation of a *Sulf1* Floxed Mouse

**FIGURE 3:**
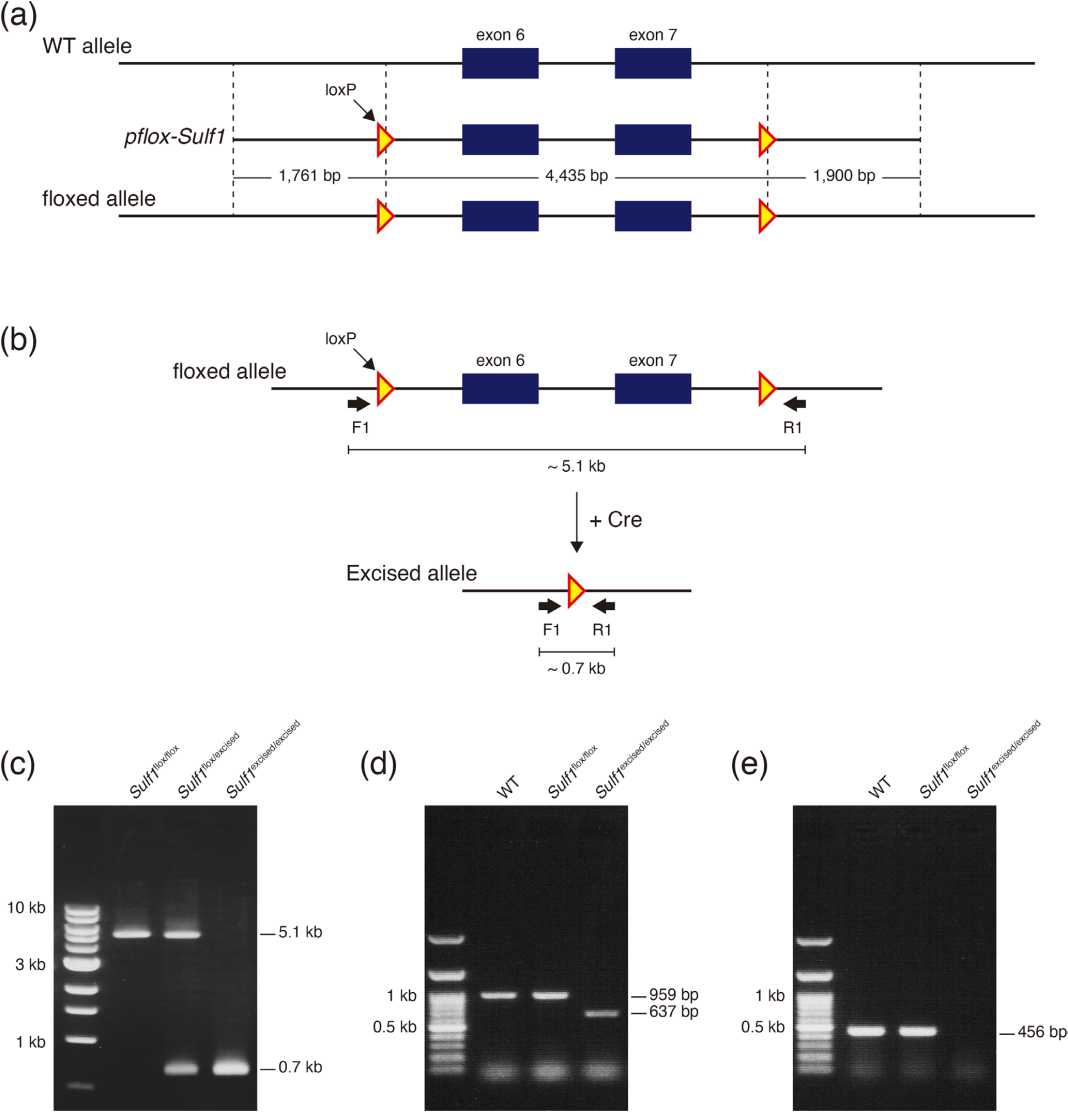
Generation of *Sulfatase 1* (*Sulf1*) floxed mouse by means of CRISPR‐Cas9‐mediated gene editing. (a, b) Schematic diagram of the *Sulf1* floxed and excised alleles. The exons 6 and 7 of the *Sulf1* gene are flanked by two loxP sequences. In the presence of Cre recombinase, exons 6 and 7 are excised. Yellow triangles indicate loxP sequences. Black arrows indicate the PCR primers for screening and genotyping. (c) A representative agarose gel image of genotyping PCR showing the floxed (5.1 kb) and excised (0.7 kb) alleles in *Sulf1* ^flox/flox^, *Sulf1* ^flox/excised^, *Sulf1* ^excised/excised^ mice. (d, e) RT‐PCR of *Sulf1* in the whole brain. When the primers in the exon 5 and exon 10 were used, *Sulf1* transcript lacking exons 6–7 (637 bp) was detected weakly in the *Sulf1* ^excised/excised^ mice (d), indicating that exons 6–7 was completely deleted but a small amount of *Sulf1* mRNA remained in the *Sulf1* excised mouse. When the primers in the exon 4 and 6 were used, no bands were detected in the *Sulf1* ^excised/excised^ mice (e).

It has been reported that the D1‐MSNs and D2‐MSNs in the NAc play distinct roles in adaptive behaviors (Hikida et al. 2010; Hikida et al. 2016; Sandoval‐Rodríguez et al. 2023; Walle et al. 2024). Therefore, we set out to determine the potential roles of *Sulf1* in D1‐MSNs and D2‐MSNs separately by use of *Sulf1* cKO mice. For this purpose, we newly generated *Sulf1* floxed mice by means of CRISPR‐Cas9‐mediated genome editing (Hsu et al. 2014). As shown in Figure 3a, two loxP sequences were inserted into the region flanking exons 6 and 7 of the *Sulf1* gene. RT‐PCR analysis demonstrated that *Sulf1* mRNA expression in the adult brain did not differ between floxed mice (*Sulf1* ^flox/flox^) compared with WT controls (Figure 3d,e), indicating that insertion of the two loxP sequences did not alter *Sulf1* expression. Cre recombinase expression deletes the *Sulf1* sequences flanked by the two loxPs to create an excised allele (Figure 3b). To examine whether Cre‐mediated excision occurs in vivo, we crossed *Sulf1* ^flox/flox^ mice with *Nestin*‐*Cre* mice and found that a few offspring mice had undergone germline recombination rather than brain‐specific recombination (Figure 3c) as reported previously (Luo et al. 2020; McLeod et al. 2020). By taking advantage of this unexpected recombination, we examined *Sulf1* mRNA expression in the adult mice in which the *Sulf1* gene was disrupted. In the excised mice, the *Sulf1* transcript lacking exons 6–7 was detected at lower levels compared with the transcript containing exons 6–7 in the control mice (Figure 3d). When the primers in exons 4 and 6 were used for PCR, no *Sulf1* transcripts were detected (Figure 3e), indicating that the sequence flanked by the two loxPs was completely excised. Given that the deletion of exons 6 and 7 gives rise to a premature termination codon in exon 8, Sulf1 protein with enzymatic activity is not produced from the excised allele despite the presence of such incomplete transcripts.

### 3.3 Cell‐Type Specific Ablation of the *Sulf1* Gene

We thus mated the *Sulf1* floxed mice with *Drd1*‐*Cre* and *Drd2*‐*Cre* mice (Gong et al. 2003; Heintz 2004) to selectively disrupt *Sulf1* in D1‐MSNs and D2‐MSNs, respectively. The resultant mice, *Sulf1* ^flox/flox^; *Drd1*‐*Cre* and *Sulf1* ^flox/flox^; *Drd2*‐*Cre* mice, are henceforth denoted as D1cKO and D2cKO mice, respectively. We examined *Sulf1* mRNA expression in brain sections by means of RNAscope fluorescent in situ hybridization. To detect *Sulf1* mRNA, we made a custom‐designed *Sulf1*‐specific probe set that targets the sequence of exons 6–7 instead of using the *Sulf1* probe set prefabricated by the manufacturer because the latter may produce high background signals owing to the presence of the *Sulf1* transcript lacking exons 6–7 even after Cre‐mediated excision.

**FIGURE 4:**
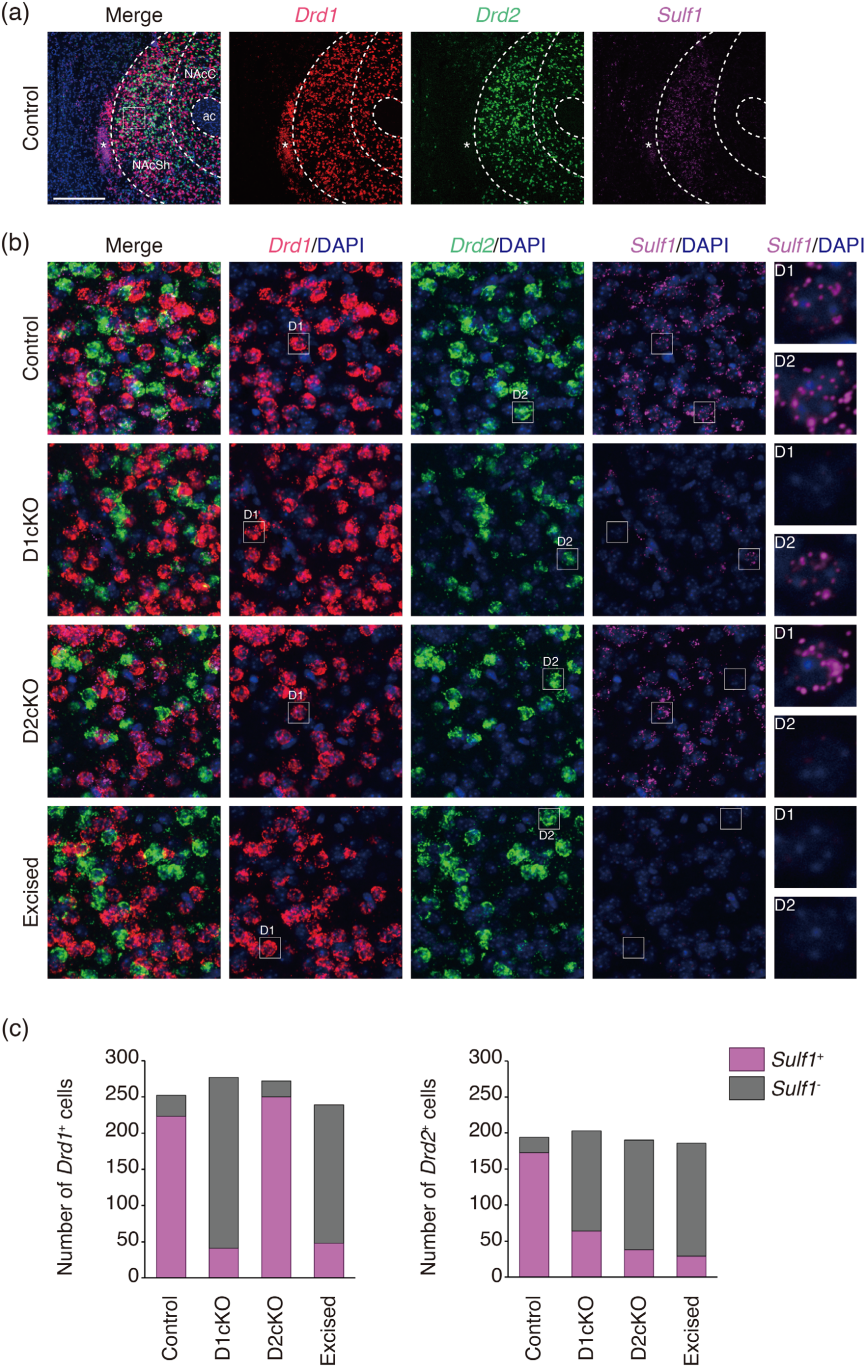
Multiplex in situ hybridization. (a) Representative images of fluorescence in situ hybridization for *Drd1* (red), *Drd2* (green), and *Sulf1* (magenta). Coronal sections containing the nucleus accumbens (NAc) are shown. Broken lines indicate the border of the NAc shell (NAcSh), NAc core (NAcC), and anterior commissure (ac). Asterisks indicate the major island of Calleja. (b) Magnified images of the boxed region in (a). Microscopic images of the *Sulf1* ^flox/flox^ (Control), *Sulf1* ^flox/flox^;*Drd1*‐*Cre* (D1cKO), *Sulf1* ^flox/flox^;*Drd2*‐*Cre* (D2cKO), and *Sulf1* ^excised/excised^ (Excised) mice are shown. The rightmost panels are the magnified images of the boxed regions in the left panels showing the *Sulf1* signals in the *Drd1* ^+^ or *Drd2* ^+^ cells. (c) Numbers of *Drd1* ^+^ and *Drd2* ^+^ cells. Sum of the positive cell numbers in 6 ROIs (160‐μm square) in each of the 4 strains, each containing 3 mice, are shown. *Drd1/Drd2*‐double positive cells are included in the count. In each bar graph, the numbers of the *Sulf1* ^+^ and *Sulf1* ^−^ cells are shown by magenta and gray, respectively. Total numbers of cells analyzed are 494 for Control, 530 for D1cKO, 509 for D2cKO, and 465 for Excised. Scale bars, 400 μm (a), 50 μm (b, left), 12 μm (b, rightmost).

We first examined *Sulf1* mRNA expression in the NAc of adult mice (Figure 4a). In the control *Sulf1* ^flox/flox^ mice, *Sulf1* was detected mainly in the NAc shell, whereas *Drd1* and *Drd2* were strongly detected in both the shell and core of the NAc as reported previously (Miya et al. 2021; Miya et al. 2025). The percentages of *Drd1/Drd2* double‐positive cells in the NAc shell were 4.7% in the control mice (*n* = 23/494), which are comparable to those in previous studies (Kupchik et al. 2015; Miyasaka et al. 2025). At high magnification, *Sulf1* signals overlapped with the *Drd1* and *Drd2* signals and *Sulf1* was expressed in most *Drd1* ^+^ and *Drd2* ^+^ cells (Figure 4b). We next observed *Sulf1* expression in the *Sulf1* cKO mice on a cell‐by‐cell basis while referring to *Drd1* ^+^ and *Drd2* ^+^ expression. In D1cKO mice, *Sulf1* signals were abolished in most *Drd1* ^+^ cells, but still detected in *Drd2* ^+^ cells (Figure 4b). On the contrary, in D2cKO mice, *Sulf1* signals were abolished in the *Drd2* ^+^ cells, but maintained in *Drd1* ^+^ cells (Figure 4b). In the excised mice, which were used as a control to demonstrate the hybridization signals when exons 6–7 were deleted, *Sulf1* signals were abolished in both *Drd1* ^+^ and *Drd2* ^+^ cells (Figure 4b). Unexpectedly, however, upon closer observation, we found that *Sulf1* signals appeared to be decreased in some *Drd2* ^+^ cells of D1cKO mice (Figure 4b). We thus decided to quantify *Sulf1* expression in *Drd1* ^+^ and *Drd2* ^+^ cells.

For this purpose, we set the appropriate background levels for *Sulf1* signals and determined the overlap between *Sulf1* and *Drd1/Drd2* using the images with background signals removed (see Materials and Methods for detail). As shown in Figure 4c, *Sulf1* was positive in most *Drd1* ^+^ cells and *Drd2* ^+^ cells of the control mice (88.7% ± 2.7% positive in *Drd1* ^+^ cells and 89.5% ± 1.6% positive in *Drd2* ^+^ cells), whereas *Sulf1* was negative in most *Drd1* ^+^ cells and *Drd2* ^+^ cells of the excised mice (21.0% ± 3.9% positive in *Drd1* ^+^ cells and 17.2% ± 6.7% positive in *Drd2* ^+^ cells). The *Sulf1* signals in the excised mice may be derived from hybridization of the probe to the incomplete *Sulf1* transcript lacking exons 6 and 7, because our custom‐designed *Sulf1* target probe contained short overhang extensions in exons 5 and 8 to fulfill the criteria of the probe length recommended by the manufacturer. Hybridization with the incomplete *Sulf1* transcript could lead to overestimation of *Sulf1* expression in the cKO mice and affect the accuracy of the cell‐type specific analysis. Despite this limitation, the analysis revealed that in D2cKO mice, *Sulf1* was positive in most *Drd1* ^+^ cells (91.7% ± 1.8% positive) and negative in most *Drd2* ^+^ cells (20.4% ± 2.2% positive), whereas in D1cKO mice, *Sulf1* was negative in most *Drd1* ^+^ cells (14.8% ± 1.4% positive) and largely decreased in *Drd2* ^+^ cells (32.0% ± 6.4% positive). These results suggest that Cre‐mediated *Sulf1* deletion occurred selectively in *Drd2* ^+^ cells in D2cKO mice but occurred in *Drd2* ^+^ cells at high frequency in addition to in *Drd1* ^+^ cells in D1cKO mice.

### 3.4 CPP Is Impaired in D1cKO Mice and IA Is Impaired in D2cKO Mice

We then performed behavioral tests using these cKO mice and compared the results with *Sulf1* ^flox/flox^ mice as a control.

**FIGURE 5:**
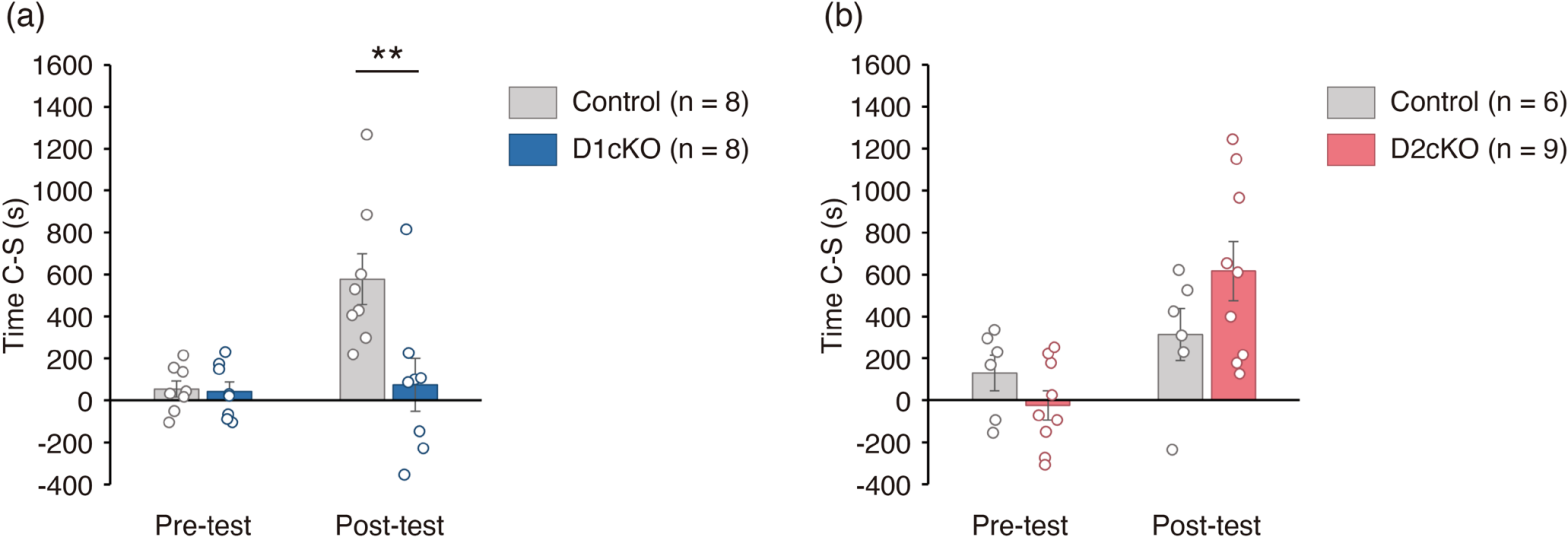
Learning in the conditioned place preference (CPP) test is impaired in D1 conditional knockout (D1cKO) mice but not in D2cKO mice. (a, b) The results of the CPP test. Time C − S was significantly lower in D1cKO mice (*n* = 8) than in control *Sulf1* ^flox/flox^ mice (*n* = 8) in the post‐test (main effect of genotype, *F*(1,14) = 6.22, *p* = 0.026; main effect of time, *F*(1,14) = 11.9, *p* = 0.0039; interaction, *F*(1,14) = 9.30, *p* = 0.0087; Bonferroni post hoc test, Control vs. D1cKO in post‐test, *p* = 0.0013; two‐way mixed model analysis of variance [ANOVA]). Time C − S did not differ significantly between control (*n* = 6) and D2cKO mice (*n* = 9; main effect of genotype, *F*(1,13) = 0.699, *p* = 0.42; main effect of time, *F*(1,13) = 9.27, *p* = 0.0094; interaction, *F*(1,13) = 2.87, *p* = 0.11; two‐way mixed model ANOVA). Individual data points and means ± SEMs are shown. **p < 0.01.

In the CPP test, Time C–S after the conditioning increased in control *Sulf1* ^flox/flox^ mice, but not in D1cKO mice (Figure 5a). A Bonferroni post hoc test revealed that the difference in Time C–S in the post‐test between the control and D1cKO mice was significant. In contrast, Time C–S did not differ between the control and D2cKO mice after the conditioning (Figure 5b).

**FIGURE 6:**
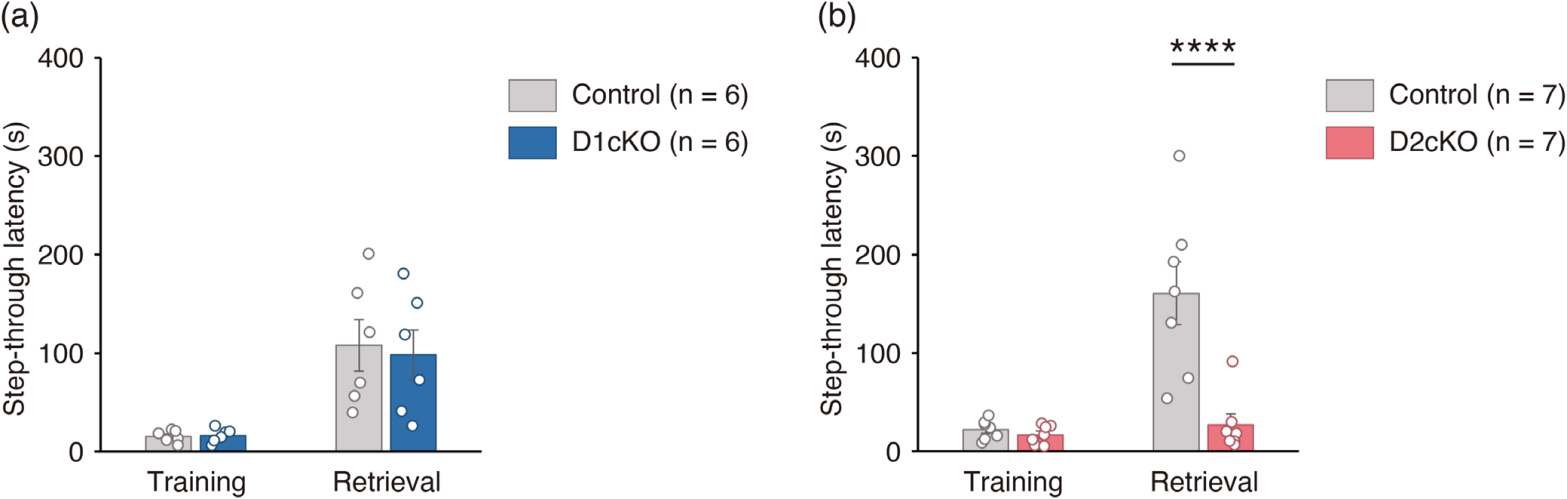
Learning in the inhibitory avoidance (IA) test is impaired in D2 conditional knockout (D2cKO) mice but not in D1cKO mice. (a, b) The results of the IA test. Step‐through latency was significantly lower in D2cKO mice (*n* = 7) than in control *Sulf1* ^flox/flox^ mice (*n* = 7) after training (main effect of genotype, *F*(1,12) = 17.5, *p* = 0.0013; main effect of time, *F*(1,12) = 17.9, *p* = 0.0012; interaction, *F*(1,12) = 13.5, *p* = 0.0032; Bonferroni post hoc test, Control vs. D2cKO, *p* < 0.0001; two‐way mixed model analysis of variance [ANOVA]). Step‐through latency after electrical shock did not differ significantly between control (*n* = 6) and D1cKO mice (*n* = 6; main effect of genotype, *F*(1,10) = 0.057, *p* = 0.82; main effect of time, *F*(1,10) = 23.7, *p* = 0.0007; interaction, *F*(1,10) = 0.086, *p* = 0.78; two‐way mixed model ANOVA). Individual data points and means ± SEMs are shown. ****p < 0.0001.

In the IA test, the step‐through latency in the retrieval test was lower in D2cKO mice than in control mice and a Bonferroni post hoc test revealed that the difference was statistically significant (Figure 6b). In contrast, the step‐through latency did not differ between control and D1cKO mice (Figure 6a).

Taken together, these data suggest that *Sulf1* expression in *Drd1*‐expressing cells was required for reward learning in the CPP test and that *Sulf1* expression in the *Drd2*‐expressing cells was required for aversion learning in the IA test.

## 4 Discussion

In this study, by using constitutive *Sulf1* KO mice, we demonstrated that *Sulf1* is required for learning in both the CPP and IA tests. Moreover, we generated a *Sulf1* floxed mouse and showed that conditional KO of the *Sulf1* gene in *Drd1*‐ and *Drd2*‐expressing neurons led to the impairment of learning in the CPP test and IA tests, respectively. These findings suggest that *Sulf1* plays critical roles in reward and aversion learning through distinct dopamine receptor‐specific neural circuits.

Our RNAscope analysis of *Sulf1* and *Drd1/Drd2* mRNA in the NAc shell revealed that *Sulf1* mRNA was markedly reduced in D1‐MSNs of D1cKO mice and D2‐MSNs of D2cKO mice. However, unexpectedly, we found that *Sulf1* mRNA was decreased in a considerable proportion of D2‐MSNs in D1cKO mice. This may be caused by leaky Cre recombination that has occurred during development in cells destined to become D2‐MSNs. Or it might be possible that *Sulf1* disruption in D1‐MSNs reduces *Sulf1* expression in D2‐MSNs in the adult brain by an unknown mechanism. The *Drd1‐Cre* mouse line used in this study was generated by the GENSAT project and is commonly used in neuroscience research (Gong et al. 2003; Heintz 2004), but no such effects outside D1R‐expressing cells have been reported. Therefore, it is necessary to pay attention to whether unexpected changes occur when this line is used to make cKO mice. Nonetheless, in D1cKO mice, *Sulf1* expression remained in 32% of D2‐MSNs and specific effects were observed in the behavioral tests. It is therefore considered that the impairment in the CPP test is attributable to *Sulf1* disruption in D1‐MSNs because *Sulf1* disruption in D2‐MSNs (namely D2cKO mice) had no effect on the CPP test. However, it is impossible to completely exclude the possibility that *Sulf1* disruption in some D2‐MSNs in addition to D1‐MSNs is required to impair learning in the CPP test. Conversely, the reason the IA test was not impaired in D1cKO mice may be explained by the possibility that learning can occur if *Sulf1* expression is present in a subpopulation of D2‐MSNs.

Our present data clearly demonstrate that *Sulf1* is required for both reward and aversion learning. Few studies have analyzed *Sulf1* functions in the adult brain, with one paper reporting that constitutive *Sulf1* KO mice showed a reduced spine density and impaired long‐term potentiation in the hippocampus, an effect attributed to developmental defects as *Sulf1* mRNA is not detected in the adult hippocampus (Kalus et al. 2009). In general, HSPGs regulate synapse development and maturation by modulating the localization of synaptic signaling molecules, organizing synaptic ECMs, and acting as synaptic organizing molecules (Condomitti and de Wit 2018; Kamimura and Maeda 2021). However, no association between HSPGs and NAc functions has been reported so far. Thus, our findings showing the strong connection between *Sulf1* and reward/aversion‐related behaviors are novel and intriguing. Given that *Sulf1* KO leads to changes in the membrane excitability and excitatory synaptic transmission in the NAc MSNs (Miya et al. 2025) and that the NAc is a critical structure for reward and aversion learning, neuronal dysfunction of the NAc most likely contributes to the impairment of learning in CPP and IA tests seen in *Sulf1* KO mice. However, the possibility that *Sulf1* disruption in brain regions other than the NAc, including the PFC, PVT, and TS, all of which also express *Sulf1* (Miya et al. 2021) and contain D1R‐ or D2R‐expressing neurons, also contributes to the behavioral abnormalities cannot be excluded. The contribution of the dorsal striatum is less likely because *Sulf1* mRNA is not expressed in the region except for the TS and the subventricular zone of the lateral ventricles (Miya et al. 2021). Future studies exploring *Sulf1* KO and the NAc dysfunction at the molecular and cellular levels will be required to understand the physiological roles of *Sulf1* in the adult brain. Moreover, other functional roles of *Sulf1* in brain regions outside the NAc are also anticipated and are important directions for future research.

We found that D1cKO and D2cKO mice showed impairment of learning in the CPP and IA tests, respectively. This is similar to our previous finding that reversible neurotransmission blockade of the D1‐MSNs and D2‐MSNs in the NAc resulted in selective impairment of Pavlovian reward and aversion learning, respectively (Hikida et al. 2010; Macpherson and Hikida 2018). As we used D1cKO and D2cKO mice in our study, we cannot exclude the possibility that the *Sulf1* disruption in *Drd1* and *Drd2* expressing neurons outside the NAc affects the results. However, it is likely that the behavioral defects observed in the cKO mice are at least partly the result of deficiency of the *Sulf1* gene in the NAc, because of the well‐known roles of the NAc in reward/aversion behaviors and the high similarity of our data with previous studies using neurotransmission blocking in D1‐MSNs and D2‐MSNs of the NAc. Although some previous studies reported the opposing roles of the NAc D1 and D2 pathways in behavioral controls (Hikida et al. 2010; Lobo et al. 2010), whether and how the two pathways regulate reward and aversion processing remains controversial. In the dorsal striatum, it has been generally accepted that the direct and indirect pathways composed of D1‐MSNs and D2‐MSNs, respectively, cause opposing effects on movement, reinforcement learning, and reward‐related behaviors (Cox and Witten 2019). Analogous to the dorsal striatum, it was reported that in the NAc as well, D1‐MSNs encode positive/rewarding information whereas D2‐MSNs encode negative/aversive information (Hikida et al. 2010; Lobo et al. 2010). However, such a simple dichotomy between the D1‐MSNs and D2‐MSNs does not apply to the NAc. Recent studies using calcium imaging and optogenetics support the view that the two MSN populations work together to drive both rewarding and aversive behaviors (Domingues et al. 2025; Vieitas‐Gaspar et al. 2025). In vivo recording of NAc D1‐ and D2‐MSN responses to rewarding/aversion stimuli in *Sulf1* KO mice will be required in future to elucidate the physiological roles of *Sulf1* in NAc circuitry.

This study has several important limitations. First, because we used constitutive *Sulf1* KO mice and D1cKO/D2cKO mice, we cannot fully exclude the possibility that developmental effects of *Sulf1* disruption affected the behavioral tests. However, this seems unlikely: although *Sulf1/2* double‐KO mice show corticospinal tract axon guidance defects, no gross neuroanatomical abnormalities have been reported in single *Sulf1* KO mice, including in our own analyses (Okada et al. 2017). Second, we used only adult male mice because cocaine's rewarding and locomotor effects are highly influenced by fluctuating ovarian hormones. Female mice show estrous‐cycle–dependent variability in NAc dopamine signaling and cocaine‐induced CPP (Becker and Hu 2008; Calipari et al. 2017), which could obscure the specific contribution of *Sulf1* to reward learning. Restricting our experiments to males allowed us to reduce hormonal variability and obtain clearer genotype effects. However, we acknowledge that the absence of female mice in this study is a limitation and that the observed effects may only apply to male mice. Given sex‐dependent differences in psychiatric disorders in humans (Williams et al. 2021), it will be important to examine the effects of *Sulf1* disruption in female mice in the future. Third, unexpected *Sulf1* mRNA reduction in D2‐MSNs of D1cKO mice raises concerns about the specificity of *Sulf1* disruption, and it is necessary to adopt a method that disrupts the *Sulf1* gene in a D1‐MSN‐specific manner in adult brains in the future. Fourth, because we did not perform electrophysiological analysis in this study, the relationship between behavioral abnormalities in the cKO mice and changes at the neural circuit level remains unclear.

In summary, our findings suggest essential roles for *Sulf1* in reward and aversion learning in the adult brain. It will be important to elucidate the molecular mechanism by which the lack of HS desulfation mediated by Sulf1 causes changes in neuronal functions in the adult brain. Given that the NAc is tightly associated with psychiatric disorders, including schizophrenia, depression, and drug addiction (Macpherson and Hikida 2019), it may also be intriguing to study the possible contribution of *Sulf1* to psychiatric disorders.

## Author Contributions

**Ken Miya:** conceptualization, data curation, formal analysis, visualization, writing – original draft, writing – review and editing, investigation. **Kent Ohta:** investigation, writing – original draft, writing – review and editing. **Kazuko Keino‐Masu:** conceptualization, supervision, writing – review and editing. **Takuya Okada:** conceptualization, supervision, writing – review and editing, investigation, formal analysis, data curation, visualization, writing – original draft. **Seiya Mizuno:** investigation, methodology, writing – review and editing. **Satoru Takahashi:** methodology, writing – review and editing. **Tom Macpherson:** methodology, investigation, writing – review and editing, funding acquisition. **Takatoshi Hikida:** conceptualization, data curation, formal analysis, funding acquisition, supervision, writing – review and editing, methodology. **Masayuki Masu:** conceptualization, funding acquisition, supervision, investigation, writing – original draft, writing – review and editing.

## Funding

This work was supported by JSPS KAKENHI Grants (JP21K15210 to T.M.; JP23K24205, JP23K18163, and JP25K02547 to T.H.; JP20300108 and JP25293065 to M.M.), AMED Grants (JP25wm0625322 and JP21gm1510006 to T.H.), and grants from Takeda Science Foundation (to T.H. and M.M.), Naito Foundation (to M.M.), SENSHIN Medical Research Foundation (to M.M.), and the Collaborative Research Program of Institute for Protein Research, the University of Osaka (CRa‐20‐03 to T.H.).

## Conflicts of Interest

The authors declare no conflicts of interest.

## Supporting information


**Figure S1:** Image data processing for removing background signals in RNAscope analysis.

## Data Availability

All data described in the manuscript are available upon request.
