# Supplementary material for: Essential Roles of Heparan Sulfate Endosulfatase Sulf1 in Reward and Aversion Learning Through Distinct Dopamine D1 and D2 Receptor Pathways in Male Mice
Source: J Neurochem. 2026 Jan 4;170(1):e70338. doi: 10.1111/jnc.70338 (PMC12766148; doi:10.1111/jnc.70338)
Supplement: Supplementary file 1 — Figure S1: Image data processing for removing background signals in RNAscope analysis. [file JNC-170-0-s001.pdf]

SUPPLEMENTARY MATERIALS

Essential roles of heparan sulfate endosulfatase Sulf1 in reward and aversion learning through distinct dopamine D1 and D2 receptor pathways in male mice

Ken Miya[1,2]*, Kent Ota[3]*, Kazuko Keino-Masu[1,2], Takuya Okada[1,2], Seiya Mizuno[4], Satoru Takahashi[4], Tom Macpherson[3], Takatoshi Hikida[3], and Masayuki Masu[1,2]

[1]Graduate School of Comprehensive Human Sciences, University of Tsukuba, Tsukuba, Ibaraki 305-8575, Japan

[2]Department of Molecular Neurobiology, Institute of Medicine, University of Tsukuba, Tsukuba, Ibaraki 305-8575, Japan

[3]Laboratory for Advanced Brain Functions, Institute for Protein Research, University of Osaka, Osaka 565-0871, Japan

[4]Laboratory Animal Resource Center, Transborder Medical Research Center, Institute of Medicine, University of Tsukuba, Tsukuba, Ibaraki 305-8575, Japan

*K.M. and K.O. contributed equally to this work.

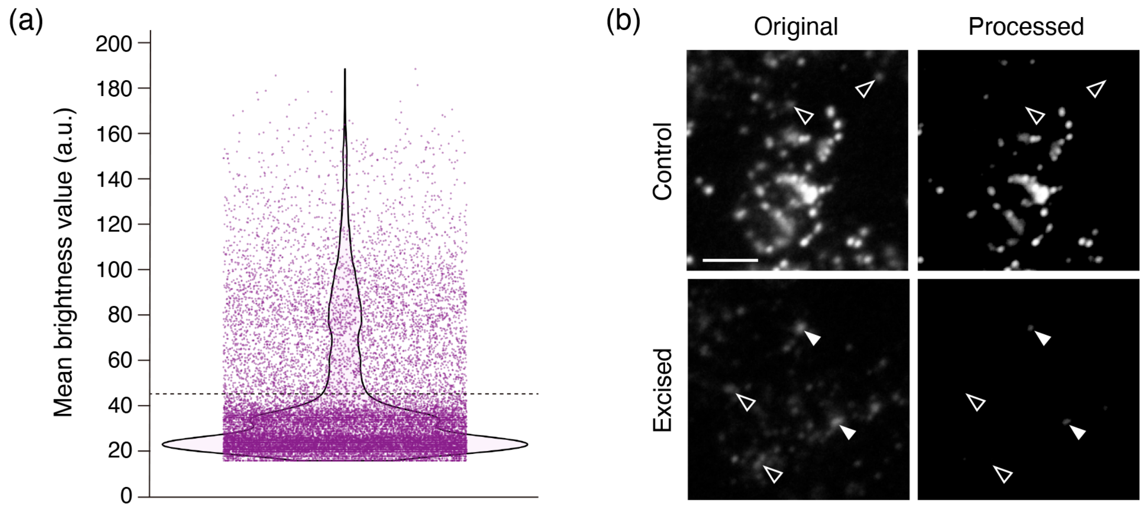

Supplementary Figure 1. Image data processing for removing background signals in RNAscope analysis.

(a) A violin plot of the mean brightness values for the *Sulf1* signals obtained by an RNAscope analysis. All the particles in the microscopic images in the *Sulf1* channels (magenta) were identified by ImageJ Fiji (https://imagej.net/ij/) and the mean brightness values for each particle were obtained by means of the Analyze Particle function. The values from all the data of all the mice examined (the control, D1cKO, D2cKO, and excised mice; a total of 24 ROIs each from 12 mice) are overlaid as dots on the violin plot. The dotted line indicates the level of 45. The signals below 45 (approximately 74% among all particles) are assumed to be background signals.

(b) Representative microscopic images before and after the background subtraction in the control and excised mice. The left panels show the original image with no data processing. The right panels show the processed images which were obtained by setting the threshold to 45. The open arrowheads indicate some of the signals that disappeared after the image processing. The closed arrowheads in the bottom panels indicate the signals that remained after image processing. Scale bar, 10 μm.
